# Oxaliplatin-induced peripheral neuropathy can be minimized by pressurized regional intravascular delivery in an orthotopic murine pancreatic cancer model

**DOI:** 10.1007/s12672-022-00483-4

**Published:** 2022-04-06

**Authors:** Jayanth Surya Narayanan Shankara Narayanan, Katie Frizzi, Suna Erdem, Partha Ray, David Jaroch, Bryan Cox, Steven Katz, Diego Vicente, Rebekah White

**Affiliations:** 1 Moores Cancer Center, University of California, 3855 Health Sciences Dr, Rm 2336, La Jolla, San Diego, CA 92037 USA; 2 Department of Pathology, University of California, San Diego, CA USA; 3 TriSalus™ Life Sciences, Inc, Westminster, CO USA; 4 Immuno-Oncology Institute, Roger Williams Medical Center, Providence, RI USA; 5 Uniformed Services University of the Health Sciences, Bethesda, MD USA

## Abstract

**Purpose:**

There is a great need to reduce the toxicity of chemotherapy used in the management of pancreatic ductal adenocarcinoma (PDAC). Here we explore if regional pressurized delivery of oxaliplatin can minimize peripheral neuropathy in mice.

**Methods:**

We used an orthotopic PDAC mouse model and delivered a single dose of oxaliplatin through the portal vein using a pressure-enabled system (pancreatic retrograde venous infusion, PRVI). We analyzed the effects of PRVI on tumor burden and peripheral neuropathy using histopathological and functional assays.

**Results:**

Tumor weights in mice treated with 2 mg/kg oxaliplatin using PRVI were significantly lower than in mice treated with the same dose systemically. This resulted in reduced peripheral neuropathy signatures in PRVI mice compared to the 20 mg/kg systemic dose required to achieve similar tumor control.

**Conclusion:**

Regional delivery of highly cytotoxic agents using PRVI can reduce the therapeutic dose of these drugs, thereby lowering toxic side effects.

## Introduction

Pancreatic Ductal Adenocarcinoma (PDAC) is one of the most aggressive major cancers and usually presents with locally advanced and/or metastatic disease [1]. Consensus guidelines are that patients with good performance status should receive systemic chemotherapy as their first line of therapy [2]. The current “optimal” regimens are either gemcitabine with nab-paclitaxel or FOLFIRINOX (folinic acid, 5-FU, irinotecan, and oxaliplatin) [3]. The highest objective response rates have been seen in studies of FOLFIRINOX but are in the range of only 20–40% [4], and this regimen is associated with high toxicity [5]. FOLFIRINOX is notable for its high incidence of grade 3 to 4 adverse events including neutropenia (45.7 vs. 21.0%), febrile neutropenia (5.4 vs. 1.2%), thrombocytopenia (9.1 vs. 3.6%), diarrhea (12.7 vs. 1.8%), and peripheral neuropathy (9 vs. 0%) compared with gemcitabine alone [5]. The higher peripheral neuropathy rate associated with FOLFIRINOX is secondary to the systemic delivery of oxaliplatin, a platinum-based cytotoxic drug which causes peripheral nerve damage [6].

Given these toxicities, alternative regional delivery methods have been developed to limit exposure of drugs to the systemic circulation and enhance tumor uptake and accumulation. Several studies over the past few decades have compared selective arterial delivery to intravenous delivery in PDAC and demonstrated improved response rates to a variety of agents [7]. This technique has evolved over the years, and the armamentarium of regional arterial delivery includes celiac axis infusion alone or in combination with selective embolization and isolated hypoxic perfusion [8]. Beyond PDAC, regional hepatic arterial delivery of cytotoxic therapy has been is relatively widespread use for colorectal liver metastases [9], and a recent clinical trial demonstrated that Pressure Enabled Drug Delivery (PEDD) of chimeric antigen receptor T-cells (CAR-T) via the hepatic artery resulted in promising treatment responses in liver metastases resistant to systemic treatment [10]. Further, the combination of pressurized arterial delivery of CAR-T and internal radiation achieved tumor growth reduction in 4 out of 6 patients, with 5 out of 6 showing reduced expression of serum markers such as CA-19-9 and minimal side-effects [11]. In a patient with liver metastases secondary to PDAC, three doses of anti-CEA CAR-T were regionally delivered via hepatic artery infusion (HAI) using PEDD technology; durable and sustained metabolic responses were observed in the liver for 13 months [10]. The PEDD method has also been evaluated for delivery of anti-PD-1 checkpoint blockade to colorectal liver metastases in a preclinical murine portal vein delivery model with demonstration of enhanced tumor control compared to systemic delivery [12].

In a recent study using a syngeneic orthotopic PDAC mouse model, the previously described PEDD method was modified to deliver gemcitabine by Pancreatic Retrograde Venous Infusion (PRVI) to the pancreatic tumor [13]. In this model, retrograde venous infusion through the portal vein with pancreatic venous isolation allowed for gemcitabine delivery to PDAC tumors at pressures 20–30 mmHg above baseline intra-tumoral pressures and resulted in significantly higher tumor uptake of the cytotoxic drug as well as improved tumor response rates. Based on this regional delivery literature, we hypothesize that PRVI of oxaliplatin in an orthotopic mouse model of PDAC would improve tumor response rates while limiting the neurotoxic side effects of the drug.

## Materials and methods

### Cell culture

The murine PDAC cell line KPC4580P, a gift from Dr. Jen Jen Yeh of University of North Carolina, was established from male LSL-Kras^G12D/+^; LSL-Trp53^R172H/+^; Pdx1^Cre/+^; LSL-Rosa26 ^Luc/+^ mouse as previously described [14]. The cells were cultured with DMEM: F12 media in 50:50 ratio with 10% FBS and 1% penicillin–streptomycin antibiotics at 37 °C with 5% CO_2_. Cells were used within three passages of thawing between experiments.

### Animal experiments

All animal experiments were approved by the Institutional Animal Care and Use Committee (IACUC) of University of California, San Diego (UCSD). All methods involving animals were performed according to Office of Laboratory Animals Welfare (OLAW)—NIH guidelines, in a facility fully accredited by the Association for Assessment and Accreditation of Laboratory Animal Care, International (AAALAC). For all experiments, wild type (WT) 12–14 week old adult male C57BL/6 (Jackson Laboratories, Bar Harbor, ME) were utilized in an orthotopic pancreatic tumor model as previously described [15]. Briefly, 500,000 KPC-4580P cells were injected SQ and allowed to grow for 10 days. The tumors were harvested, and small pieces (~ 2 mm^3^) were implanted onto the tail of the mouse pancreas. Orthotopic tumor growth was monitored using ultrasound evaluation (*SonoQue L5P* handheld ultrasound) until tumors reached 5 mm in diameter. To determine the maximum tolerated dose (MTD) of oxaliplatin, tumor-bearing mice received systemic oxaliplatin infusion at concentrations of 0, 4, 12, 20 and 40 mg/kg (n = 5/group) and monitored for 7 days. For treatment experiments, mice underwent PRVI as previously described [13]. In short, PRVI oxaliplatin and PRVI saline mice underwent laparotomy followed by temporary pancreatic venous isolation with microvascular clamps (*Roboz Surgical, Boston, MA*), and portal vein cannulation. Mice were randomized to receive either oxaliplatin (Teva pharmaceutical, 5 mg/mL pharmaceutical grade) 2 mg/kg in 100 µL saline or saline (100 µL), infused at a rate of 5 mL per minute. The venous isolation was released after 5 min of clamp time. The portal vein puncture site was covered with Surgicel (*Ethicon, Raritan, NJ*). Intraperitoneal injections of oxaliplatin at concentrations of 2, 20, or 40 mg/kg were used as systemic controls. Mice (n = 3) from the PRVI oxaliplatin and systemic oxaliplatin groups were randomly selected for euthanasia at 3 h post-infusion to evaluate platinum concentrations within tumors as well as in plasma. All mice were euthanized using slow fill CO_2_ asphyxiation (1.5 L/min) followed by cervical dislocation on day 7, and tumors and foot skin (peripheral nerves) were harvested and evaluated for treatment response.

### Histopathological analysis

Tissue (foot skin) specimens collected from euthanized mice 7 days after infusion underwent histologic evaluation. Tissues were fixed in 10% neutral buffered formalin and dehydrated with ethanol followed by embedding in paraffin cassettes. Sections (6 µm thick) of foot skin were cut using a Leica manual rotary microtome and mounted as 2 serial sections per slide. For IHC staining using the neuronal marker PGP 9.5, slides were deparaffinized with xylene, hydrated using ethanol gradient, and quenched with methanol/hydrogen peroxide, followed by antigen retrieval with citrate buffer (pH=6). Tissue sections were blocked with horse serum and incubated with primary antibody anti−mouse PGP 9.5 (ProteinTech) followed by HRP−conjugated secondary antibody. DAB (3,3’−diaminobenzidine) solution was used to develop the staining and counterstained with hematoxylin. The slides were imaged using an Olympus SC100 microscope at 20×magnification. Tissue parameters such as length of tissue section and regions of interest were defined using Qupath 3.0 software. The number of PGP 9.5 stained foci were manually counted independently within the epithelium and the dermal layers as Intra−Epidermal Nerve Fibers (IENF) and Sub−epidermal Neural Plexi (SNP), respectively in 5 mice/group with each data point representing the mean value from 3 regions of interest per mouse.

### Measurement of platinum concentration

Tumor tissue from animals was collected into 15 mL conical tubes, documenting actual tissue weights, and washed with 70% ethanol. In the fume hood, 571 µL of Metal Free Nitric Acid (37%) was added to the tube containing the tissue and vortexed at high speed for 10 s. Samples were left in a fume hood overnight for tissue digestion. Once tissue was fully dissolved, the total weight of the mixture was determined and increased to a final volume of 10 mL with ultrapure distilled water. Samples were centrifuged and the supernatant was filtered and collected for Inductively Coupled Plasma−Mass Spectroscopy analysis. The ICP−MS analysis was done on a Thermo Scientific iCAP RQ ICP−MS in the Environmental and Complex Analysis Laboratory on the UC San Diego campus. The analysis was conducted in standard (STD) mode monitoring 159Tb and 209Bi as internal standards. The results were reported as ppb of platinum relative to the initial tumor weight [16].

### Motor nerve conduction velocity

Mice were anesthetized using 2.5–4 ppm isoflurane in oxygen and transferred to a circulating water–heated pad, with anesthesia maintained via a face mask connected to the isoflurane regulator. Two recording electrodes (platinum−tipped sub−dermal needle electrodes, Grass Technologies) were inserted into the interosseous muscles between the animal’s second, third, and fourth toes, and secured to the heating pad with lab tape. A grounding electrode was placed into the skin at the neck. A PowerLab stimulator (AD instruments) was set to deliver a 200 mV, 50 μs−duration square−wave stimulus every 2 s. The stimulating electrode was inserted into the ankle near the Achilles tendon and adjusted until the resulting M waves were clear and maximal. The electrode was removed and inserted into the sciatic notch at the hip and resulting M waves were recorded, as previously described [17]. The distance between the stimulation sites at the hip and ankle was measured and divided by the time (latency) between the peaks of proximal and distal sites to obtain the motor nerve conduction velocity (MNCV).

### Statistical analysis

All data were analyzed using GraphPad Prism 8.0 software (GraphPad Software Inc, La Jolla, CA). All tumor and peripheral nerve data are represented as mean ± standard error of the mean (SEM). Student’s *t* test with 2-tailed hypothesis was used to compare 2 groups or 1-way analysis of variance with Tukey’s multiple comparison test was used for multiple groups. Survival was compared between groups by Kaplan–Meier analysis.

## Results

### Induction of peripheral neuropathy in mice after acute systemic oxaliplatin exposure

**Fig. 1:**
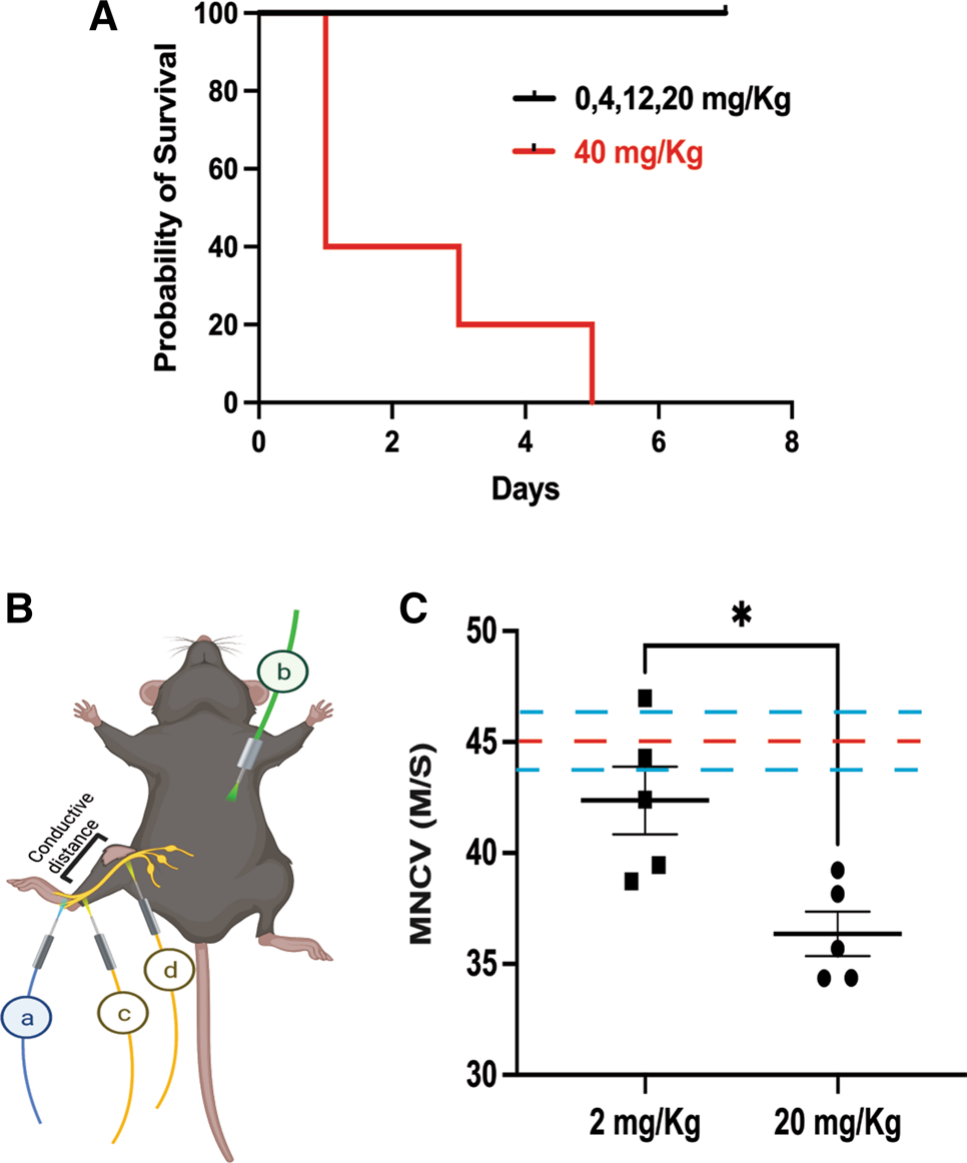
Acute exposure of high dose systemic oxaliplatin can cause peripheral neuropathy in mice. **A** Kaplan Meier survival analysis of C57BL/6 harboring orthotopic pancreatic tumors exposed to different systemic (IP) doses of oxaliplatin shows the dose of 40 mg/kg causing acute toxicity and death in this model. **B** Motor Nerve Conduction Velocity (MNCV) measurement shows a reduction of nerve function. Schematic of the MNCV experiment on mice showing the placement of recording electrode in the intraosseous muscles electrode (**a**), grounding electrode **b** and the stimulating electrodes at the ankle **c** and sciatic notch. Time taken for the nerve impulse to travel c to d was measured to calculate the MNCV. **C** MNCV data represented as mean±SEM of 5 mice per group of systemic oxaliplatin delivery with each data point representing the mean value from 3 repeated measurements per mouse. Red dotted line represents the average MNCV of untreated mice with 95% confidence intervals (blue dotted lines) reported elsewhere [19]

Peripheral neuropathy is a known side effect of chronic exposure to oxaliplatin over several weeks to months [18]. However, to establish an acute model of peripheral neuropathy in mice, we exposed KPC4580P orthotopic pancreatic tumor−bearing C57BL/6 mice to escalating single doses of oxaliplatin to observe changes in peripheral nerves within a seven−day period. The dose of 40 mg/kg was highly toxic to the mice resulting in greater than 90% death within five days of injection (Fig. 1A). Doses of 20 mg/kg and lower were well tolerated by the mice without signs of systemic toxicity. To further confirm the peripheral neuropathic effects of systemic oxaliplatin, motor nerve conduction velocity (NCV) measurements were performed (Fig. 1B). Compared to the historical average of C57BL/6 mice of this age [19] (red dotted line with 95% confidence interval in blue, Fig. 1C), the average MNCV of both the 20 and 2 mg/kg groups were lower with the 20 mg/kg dose group showing a significant (P< 0.01) slowing compared to the 2 mg/kg dose group. These experiments confirm the potential for oxaliplatin to generate large motor fiber damage within 7 days of exposure.

### Pancreatic Retrograde Portal Venous Infusion (PRVI) reduced tumor burden at a lower oxaliplatin dose

**Fig. 2:**
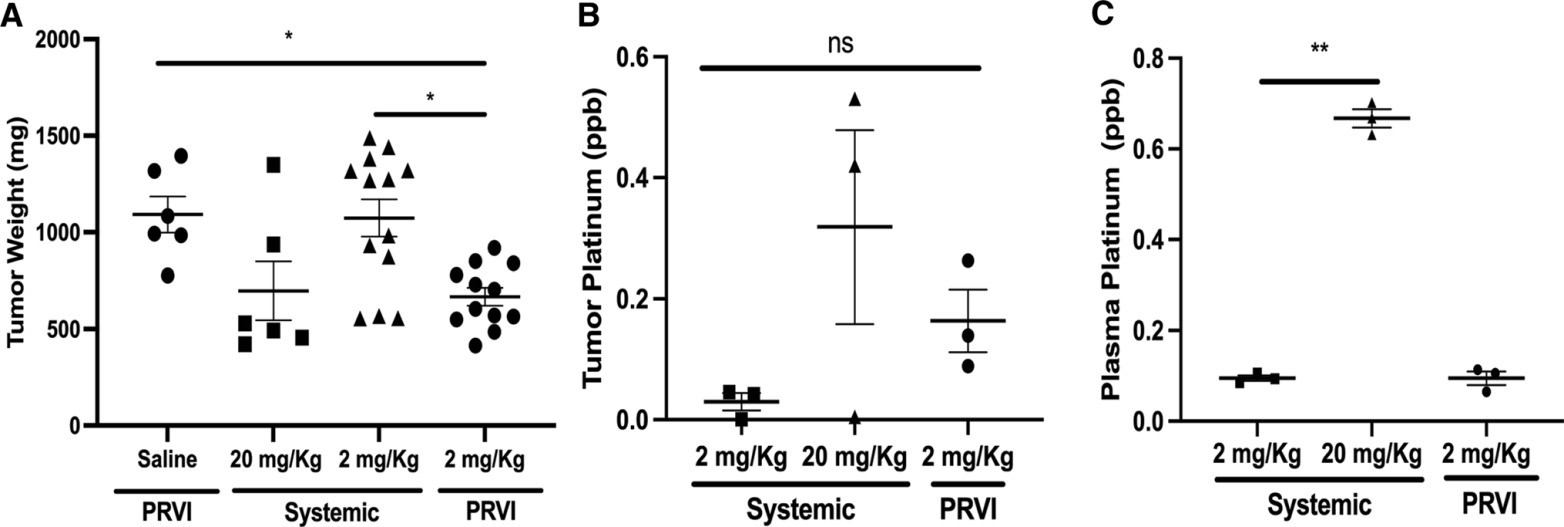
Pancreatic retrograde portal venous infusion (PRVI) of oxaliplatin can minimize the neuropathic effects of systemic oxaliplatin delivery by reducing the dose required for effective tumor control. **A** Tumor burden in the orthotopic PDAC mice 7 days post treatment shown as mean±SEM of tumor weights among the survivors of n=15 mice in each group. **B** Platinum concentration in the tumor and the circulating platinum levels in the plasma **C** were measured 3 h post infusion using inductively coupled plasma mass spectrometry (ICP−MS) and represented as mean±SEM parts per billion in 3 mice per group

To localize oxaliplatin exposure to tumors, we employed PRVI to deliver oxaliplatin retrograde through the portal vein to the pancreas. Since the maximum tolerated dose of systemic oxaliplatin was 20, 2 mg/kg was chosen as a tenfold lower and therefore safe dose for comparison of systemic and PRVI delivery. Examination of tumor burden 7 days after procedure (Fig. 2A) shows that the average tumor weight of the PRVI group (0.66±0.07 g) at a concentration of 2 mg/kg was significantly lower than the PRVI saline group (1.09±0.09 g, P< 0.05) and lower than the group of mice exposed to the same low concentration of oxaliplatin through systemic delivery (1.07±0.1 g, P< 0.05). The average tumor burden at the higher concentration of 20 mg/kg through systemic delivery (0.66±0.15 g) was not statistically different than the PRVI delivery of oxaliplatin at 2 mg/kg (P= 0.99). Exploratory analysis of platinum accumulation within a subset of tumors at 3 h post infusion showed that the PRVI oxaliplatin 2 mg/kg mice had a fourfold increased tumor accumulation of platinum compared to systemic delivery at the same concentration. By comparison, a tenfold increase in the systemic dose from 2 to 20 mg/kg of oxaliplatin generated only a twofold increase in tumor platinum concentration (Fig. 2B). Of note, these differences did not reach statistical significance due to the small sample sizes. The plasma concentration of platinum, however, was not affected by the mode of delivery, with values proportional of the initial concentration of the drug (Fig. 2C). This finding indicates that PRVI is not preventing systemic exposure to drug.

### Therapeutic systemic doses of oxaliplatin generated peripheral nerve damage

**Fig. 3:**
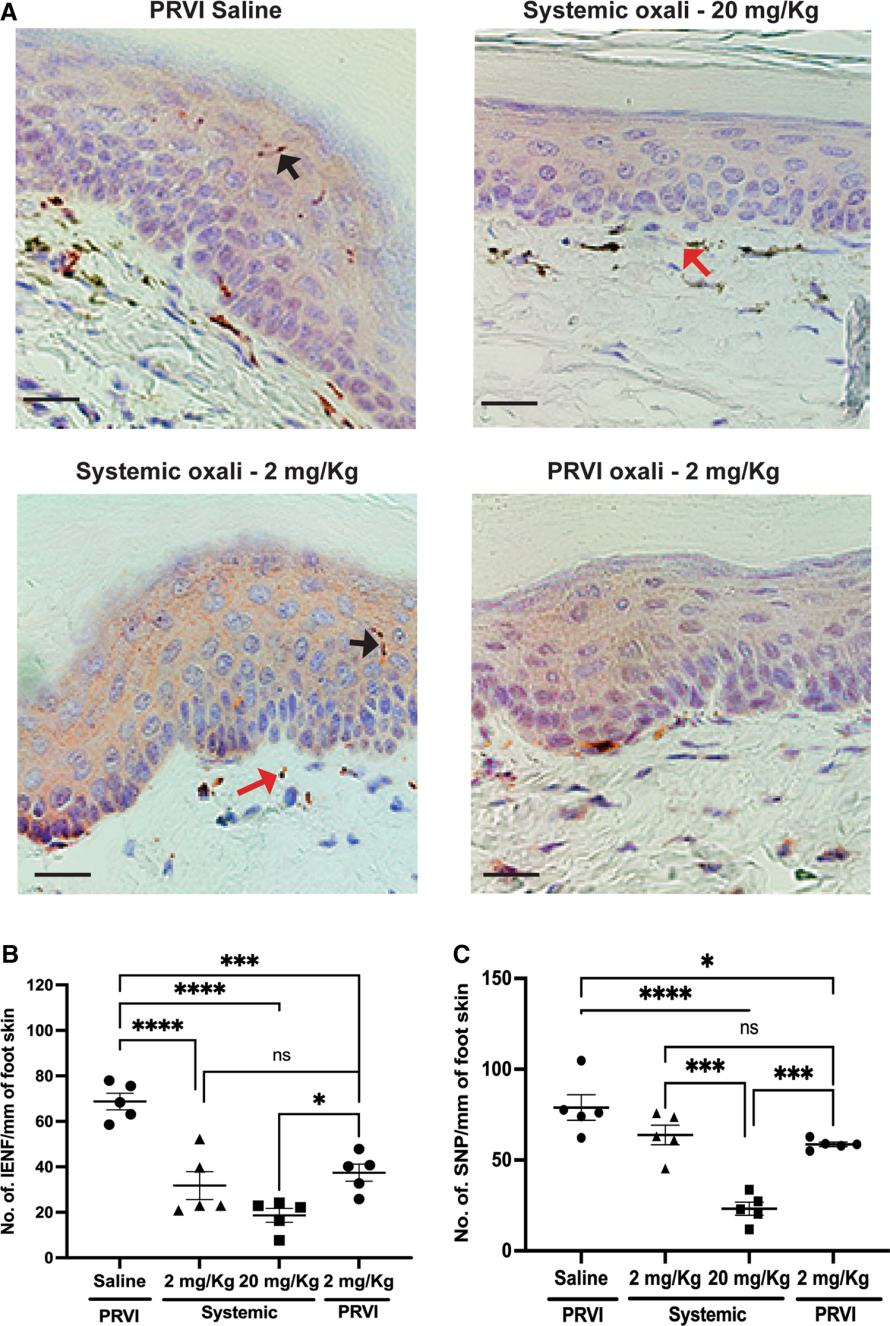
Neuropathic effects of systemic vs PRVI oxaliplatin delivery. **A** Immunohistochemical staining for the measurement of number of fibers that express PGP 9.5 neuronal marker in foot skin 7 days post oxaliplatin infusion shows the extent of neurodegeneration. Scale bar=20 µm. Black arrow indicates IENF and red indicates SNP. The density of **B** Intra−epidermal nerve fibers (IENF) and **C** Sub−epidermal Neuronal Plexi (SNP) were compared between the groups under a light microscope at 20X magnification. Data represented as mean±SEM of 5 mice/group with each data point representing the mean value from 3 regions of interest per mouse

**Fig. 4:**
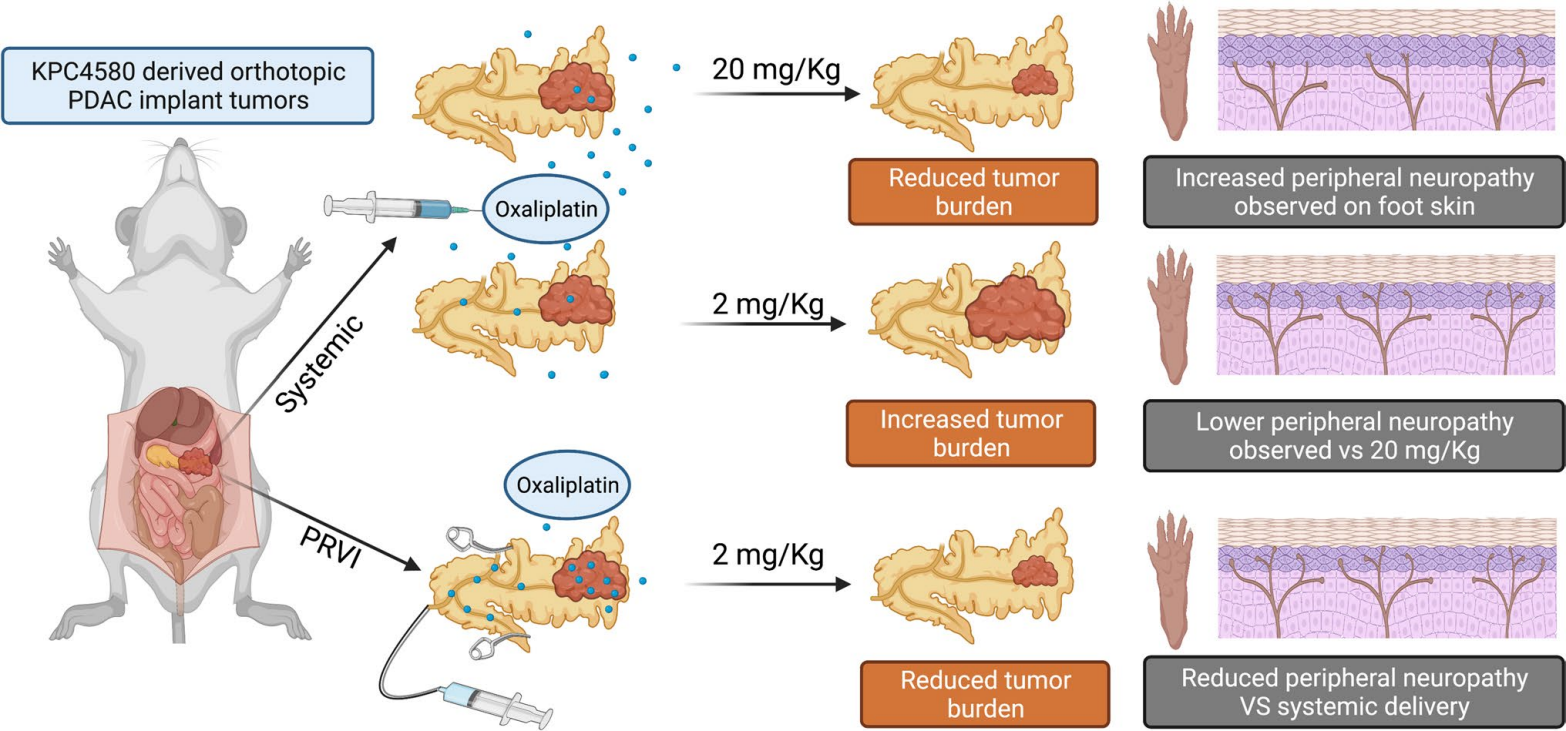
Schematic representation of the study design and outcome, created with BioRender.com. This study demonstrated that PRVI delivery of oxaliplatin achieved similar tumor control to systemic delivery with a tenfold lower dose (2 vs. 20 mg/kg). Peripheral neuropathy increased with greater systemic exposure to oxaliplatin. The risk of peripheral neuropathy can therefore be minimized with the lower therapeutic dose of PRVI

Quantification of PGP 9.5 positive nerve fibers, a pan−neuronal cytoplasmic marker, allows assessment of nerve fiber density in the foot skin of mice treated with oxaliplatin (Fig. 3A). Even the low concentration of 2 mg/kg significantly (P< 0.05) reduced the density of intra−epidermal nerve fibers (IENF) compared to the saline infusion with no significant difference between the PRVI and systemic infusion groups (Fig. 3B). There were no significant differences in sub−epidermal neural plexi (SNP) between either of the 2 mg/kg groups and the saline infusion group. However, the nerve damage seemed to be a dose−dependent phenomenon, and both the IENFs and the SNPs were reduced in the group that received 20 mg/kg compared to the other groups (Fig. 3C and 4), showing the detrimental effects of the dose of oxaliplatin necessary to affect tumor growth by systemic delivery.

## Discussion

Maximizing the efficacy of therapeutic agents while minimizing undesirable side effects is a key principle of cancer therapy. FOLFIRINOX is currently the most effective approved regimen for PDAC, but due to high rates of toxicity, it is commonly administered at 75% of the previous standard doses (“modified” or mFOLFIRINOX) [20]. Even mFOLFIRINOX, however, is associated with significant toxicity, and oxaliplatin−induced neuropathy is one of the most common treatment−limiting side effects [21] and often becomes a chronic effect [21]. The goal of regional delivery is to increase the amount of drug that is taken up by cancer cells and thereby reduce total systemic exposure to drug. Various regional intra−arterial delivery approaches via the celiac and/or gastroduodenal arteries have been described in an attempt to increase drug concentrations within pancreatic tumors relative to the systemic circulation [7]. However, these approaches rely on the limited arterial blood supply of pancreatic tumors, which is lower than the normal pancreas, liver, and other adjacent organs [22]. PRVI utilizes retrograde infusion with temporary isolation of the venous drainage system to promote effusion into the tumor. Our prior study utilizing gemcitabine in a similar mouse model directly demonstrated a transient increase in intersitial fluid pressures within tumors during PRVI [13]. In this study, we focused on oxaliplatin due to its narrow therapeutic window and the potential for regional delivery to improve it.

Limiting systemic exposure of cytotoxic drugs while maintaining effectiveness is the key to avoiding undesirable side effects. In mouse models, repeated dosing of systemic oxaliplatin at 2–4 mg/kg every 2 days over 3 weeks has been reliably associated with peripheral neuropathy [18]. Since this dosing schedule is not relevant to PRVI as it does not support repeated infusions, we sought to establish a model using a single high dose of oxaliplatin. Only one study has reported peripheral neuropathy at a 40 mg/kg dose of oxaliplatin in a survival mouse model [23], however we observed that this dose was extremely toxic to our mice model and we used maximum tolerated dose of 20 mg/kg for systemic delivery in our study. Immunohistochemical analyses of peripheral nerves in our study showed that peripheral neuropathy is a consequence of total drug exposure. The pathophysiological effects of chemotherapy−induced peripheral neuropathy exhibited by our mouse model are representative of the human disease and thus this serves as an appropriate model to evaluate the toxicity of oxaliplatin.

PRVI oxaliplatin delivery was able to reduce PDAC tumor burden at a concentration ten−fold lower than what was required through systemic delivery. Although differences in platinum accumulation did not reach statistical significance due to low sample size, we believe this is due to the ability of PRVI to achieve higher tumor accumulation of the cytotoxic agent compared to the non−pressurized systemic delivery system. In our prior studies with gemcitabine, we were able to demonstrate a sevenfold greater accumulation of gemcitabine with PRVI compared to systemic delivery at the same dose [13]. PRVI in our mouse model was not able to significantly reduce the systemic exposure of oxaliplatin, based on the similar plasma platinum levels between systemic and PRVI delivery of the same dose. However, PRVI allows use of a much lower dose of drug than needed with systemic delivery, minimizing the risk of peripheral neuropathy. Tumor growth was inhibited with a single dose of single−agent oxaliplatin at 2 mg/kg via PRVI, demonstrating that our prior findings with gemcitabine are generalizable to other cytotoxic agents. We envision that PRVI can potentially be used to deliver multi−drug regimens or in combination with systemic delivery of less toxic, synergistic agents.

One of the limitations of this study is that we could not directly show that peripheral neuropathy was avoided in mice receiving PRVI oxaliplatin due to the practical challenges of performing NCV measurements in mice recovering from a major surgical procedure. Another is that our model is not compatible with repeated dosing, since performing multiple survival PRVI surgeries on mice is not technically and ethically feasible. An FDA−approved delivery device (TriSalus Infusion System, TIS-21120-60, TriSalus Life Sciences, Westminster CO) has been designed to accomplish PRVI clinically. The device is introduced into the portal venous system by percutaneous transhepatic access though a technique similar to that used for portal vein embolization [24]. PRVI in humans can be performed much less invasively than in mice such that repeated dosing would be feasible. In patients with borderline resectable or locally advanced PDAC, a significant response to PRVI chemotherapy could potentially render them candidates for surgical resection. Ongoing preclinical studies are evaluating different combinations of cytotoxic drugs as well as immunotherapeutic agents, and a clinical trial evaluating PRVI delivery of oxaliplatin is currently in development.

## Data Availability

All data generated or analyzed during this study are included in this published article.

## References

[1] Siegel RL (2021). Cancer Statistics, 2021. CA Cancer J Clin.

[2] Tempero MA (2021). Pancreatic adenocarcinoma, version 2.2021, NCCN clinical practice guidelines in oncology. J Natl Compr Canc Netw.

[3] Kang J (2018). Nab-paclitaxel plus gemcitabine versus FOLFIRINOX as the first-line chemotherapy for patients with metastatic pancreatic cancer: retrospective analysis. Invest New Drugs.

[4] Tong H (2018). The benefits of modified FOLFIRINOX for advanced pancreatic cancer and its induced adverse events: a systematic review and meta-analysis. Sci Rep.

[5] Conroy T (2011). FOLFIRINOX versus gemcitabine for metastatic pancreatic cancer. N Engl J Med.

[6] Cersosimo RJ (2005). Oxaliplatin-associated neuropathy: a review. Ann Pharmacother.

[7] Liu F (2012). Regional intra-arterial vs. systemic chemotherapy for advanced pancreatic cancer: a systematic review and meta-analysis of randomized controlled trials. PLoS ONE.

[8] Lorenz M (2000). Regional chemotherapy in the treatment of advanced pancreatic cancer—is it relevant?. Eur J Cancer.

[9] Kemeny NE (2009). Conversion to resectability using hepatic artery infusion plus systemic chemotherapy for the treatment of unresectable liver metastases from colorectal carcinoma. J Clin Oncol.

[10] Katz SC (2020). HITM-SURE: Hepatic immunotherapy for metastases phase Ib anti-CEA CAR-T study utilizing pressure enabled drug delivery. J Immunother Cancer.

[11] Katz SC (2020). HITM-SIR: phase Ib trial of intraarterial chimeric antigen receptor T-cell therapy and selective internal radiation therapy for CEA+ liver metastases. Cancer Gene Ther.

[12] Chai LF (2019). Regional pressure-enabled drug delivery of anti-PD-1 agent for colorectal liver metastases improves anti-tumor activity without increased hepatic toxicity. J Immunol.

[13] Shankara Narayanan JS (2020). Pressure-enabled delivery of gemcitabine in an orthotopic pancreatic cancer mouse model. Surgery.

[14] Narayanan JSS (2019). irreversible electroporation combined with checkpoint blockade and TLR7 stimulation induces antitumor immunity in a murine pancreatic cancer model. Cancer Immunol Res.

[15] Shankara Narayanan JS (2018). A syngeneic pancreatic cancer mouse model to study the effects of irreversible electroporation. J Vis Exp.

[16] Li J (2014). In vivo biodistribution of platinum-based drugs encapsulated into multi-walled carbon nanotubes. Nanomedicine.

[17] Jolivalt CG (2016). Peripheral neuropathy in mouse models of diabetes. Curr Protoc Mouse Biol.

[18] Marmiroli P (2017). Susceptibility of different mouse strains to oxaliplatin peripheral neurotoxicity: phenotypic and genotypic insights. PLoS ONE.

[19] Walsh ME (2014). Use of nerve conduction velocity to assess peripheral nerve health in aging mice. J Gerontol Series A.

[20] Kang H (2018). Comparison of efficacy and safety between standard-dose and modified-dose FOLFIRINOX as a first-line treatment of pancreatic cancer. World J Gastrointest Oncol.

[21] Han CH (2016). Preventing oxaliplatin-induced neurotoxicity: rationale and design of phase Ib randomized, double-blind, placebo-controlled, cross-over trials for early clinical evaluation of investigational therapeutics. Expert Opin Drug Metab Toxicol.

[22] Liu X (2016). Gemcitabine-based regional intra-arterial infusion chemotherapy in patients with advanced pancreatic adenocarcinoma. Medicine (Baltimore).

[23] Sprowl JA (2013). Oxaliplatin-induced neurotoxicity is dependent on the organic cation transporter OCT2. Proc Natl Acad Sci.

[24] Avritscher R (2008). Percutaneous transhepatic portal vein embolization: rationale, technique, and outcomes. Semin Intervent Radiol.

